# Effect of Casting Position on Mechanical Performance of Ultra-High Performance Concrete

**DOI:** 10.3390/ma15020404

**Published:** 2022-01-06

**Authors:** Sujing Zhao, Yiheng Bo

**Affiliations:** College of Mechanics and Materials, Hohai University, Nanjing 211106, China; yihengbo6@163.com

**Keywords:** UHPC, casting, strength, fracture property

## Abstract

The mechanical performance of ultra-high performance concrete (UHPC) is a function of fiber distribution and orientation, which are affected by the processing of the fresh material. In this study, the influences of two casting positions (mid-cast and end-cast) on strength and fracture properties of UHPCs with different fiber types and fiber contents were investigated. The results show that mid-cast specimens have higher flexural strength and fracture properties than end-cast specimens, while the compressive strength is almost unaffected by casting position. Compared to specimens with straight fibers, the flexural strength of specimens with hooked-end fibers is more likely to be affected by casting position. The residual load-to-peak load ratio is independent of casting position but affected by fiber type and fiber content.

## 1. Introduction

Ultra-high performance concrete (UHPC) is a kind of advanced cementitious material with enhanced packing density, which is produced through improved homogenization, low water/binder ratio (w/b), and use of reactive powders [1]. With the combination of micro steel fibers, the material exhibits outstanding performance in terms of strength, toughness, ultimate strain, and durability [2]. Over the last twenty years, UHPC has been increasingly used in areas such as structural segments, bridge decks, pipes, cladding panels, rehabilitation, and wear protection [3,4,5,6,7].

Compared with conventional fiber reinforced concrete, UHPC is characterized by strain-hardening or deflection-hardening behavior after matrix cracking [8,9]. This superior post-cracking performance is derived from the stress transfer provided by microfibers bridging at the crack surface. The load-bearing capacity of the bridging fibers is governed by the property of fibers, matrix formulation, and fiber–matrix interactions, as well as the distribution and orientation of fibers [10,11,12]. For structural applications, the influence of fiber distribution and orientation on mechanical performance must be considered because variations in fiber distribution and orientation could lead to significant differences in mechanical properties within the large sections and thus cause structural instability [13].

Many researchers have revealed that fiber distribution and orientation can be affected by the rheology of the fresh mix, the methods of mixing used, casting, vibrating, and the geometry and dimensions of the mold, etc. Zhou and Uchida found that the flowability of UHPC dictates fiber orientation in areas close to the formwork surface and that with the increase of flowability fibers are prone to be oriented parallel to the longitudinal direction of slabs [14]. Barnett et al. investigated the influence of three different casting methods (concrete poured at the center of the panel, poured at several points around the perimeter of the panel, and poured randomly) on the fiber orientation and flexural strength of UHPC round panels, and the results indicated that the fiber tended to align perpendicularly to the direction of flow and that panels poured from the center showed much higher strength than panels poured by the other two methods [15]. Similar results were obtained by Zhou and Uchida, who reported that for a circular panel cast from the center fibers tend to orientate in concentric circles within the panel [16]. Another study adopted two different casting methods to induce a random fiber distribution and a preferred fiber distribution along the flow direction, and the results showed that the obtained tensile strength of the latter was twice that of the former [17]. In addition, Huang reported that, due to geometrical restraint effects on fiber rotation, better fiber orientation and higher flexural performance can be obtained near formwork boundaries [18,19].

Therefore, the manufacturing process can significantly influence fiber distribution and orientation in the fresh material and thus affect mechanical performance. In this respect, previous studies have mainly focused on strength properties and there are few studies that have reported effects on other mechanical behaviors, such as toughness and fracture properties. Moreover, there is a lack of research concerning the effect of fiber type on mechanical behavior when different manufacturing processes are involved. In this research, UHPC specimens were prepared using two different casting methods, i.e., middle casting (mid-cast) and end casting (end-cast), and the influence of casting position on the mechanical properties of UHPC with different fiber types and contents was investigated. The research is expected to reveal the importance of casting to material properties and to provide some insights into specimen preparation and testing.

## 2. Materials and Methods

### 2.1. Raw Materials and Mix Proportion

Portland cement with a type of P•II 52.5 (Conch, Wuhu, China) in accordance with Chinese Standard GB 175–2020 [20] and silica fume (Elkem, Shanghai, China) were used as cementitious materials. The physical and chemical properties of the two reactive powders are given in Table 1. Natural river sand (Yangtze River, Nanjing, China) with an air-dry apparent density of 2.65 g/cm^3^ and fineness modulus of 2.3 was used as aggregate, and the mixing water was tape water (Nanjing, China). In order to make the material workable at low water/binder ratio (w/b), a polycarboxylate-based superplasticizer (PCA-I, Sobute, Nanjing, China) was admixed. Moreover, micro steel fibers were added to improve the strength and toughness of the material. Three types of brass-coated steel fibers, i.e., short straight fibers (WSF0108, Daye, Ganzhou, China), long straight fibers (WSF0213, Daye, Ganzhou, China), and hooked-end fibers (GSF0325, Daye, Ganzhou, China), were used in this study and their dimensions are given in Table 2.

The mix proportion of the UHPC is given in Table 3. A relatively low aggregate-to-binder ratio and a high dose of superplasticizer were adopted to make the UHPC exhibit self-consolidating properties, so that with a fixed casting position the fresh mix could flow into every corner of the mold effortlessly. The volume fraction of steel fibers in UHPC was 1% or 1.5%. Five UHPC mixes, which have the same matrix but different fibers, were designed in this study. Based on fiber type and fiber content, the five mixes were denoted by S1, S1.5, L1, L1.5, and H1.5, where S, L, and H are the initials of the three types of fiber, and the Arabic number indicates the fiber volume content in the mix.

### 2.2. Specimen Preparation and Curing

The raw materials were mixed using a mortar mixer (UJZ-15, Lanbiao, Cangzhou, China) with a capacity of 0.015 m^3^. The cement, silica fume, and sand were first dry-mixed for 3 min to homogenize the material. Then, water and superplasticizer were gradually added before the fresh material was mixed for 5 min. Finally, fibers were spread into the flowable mixture followed by mixing for 2 min to disperse the fibers in the material.

**Figure 1:**
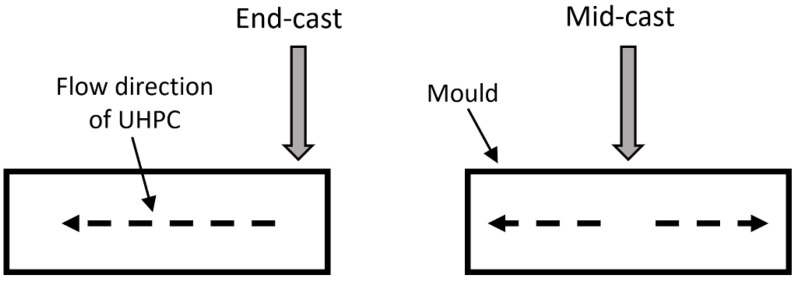
Schematic of casting at two different positions. Left: end casting (end-cast); right: middle casting (mid-cast).

After mixing, the fresh material was placed into prism molds (Huake, Shijiazhuang, China) with a dimension of 40 mm × 40 mm × 160 mm. The material was cast at either the middle or the end of the mold, as shown in Figure 1. The specimens were cured for 1 day at room temperature of about 20 °C. They were then demolded and cured in a standard moisture room to prescribed ages before the mechanical tests were performed.

### 2.3. Test Methods

**Figure 2:**
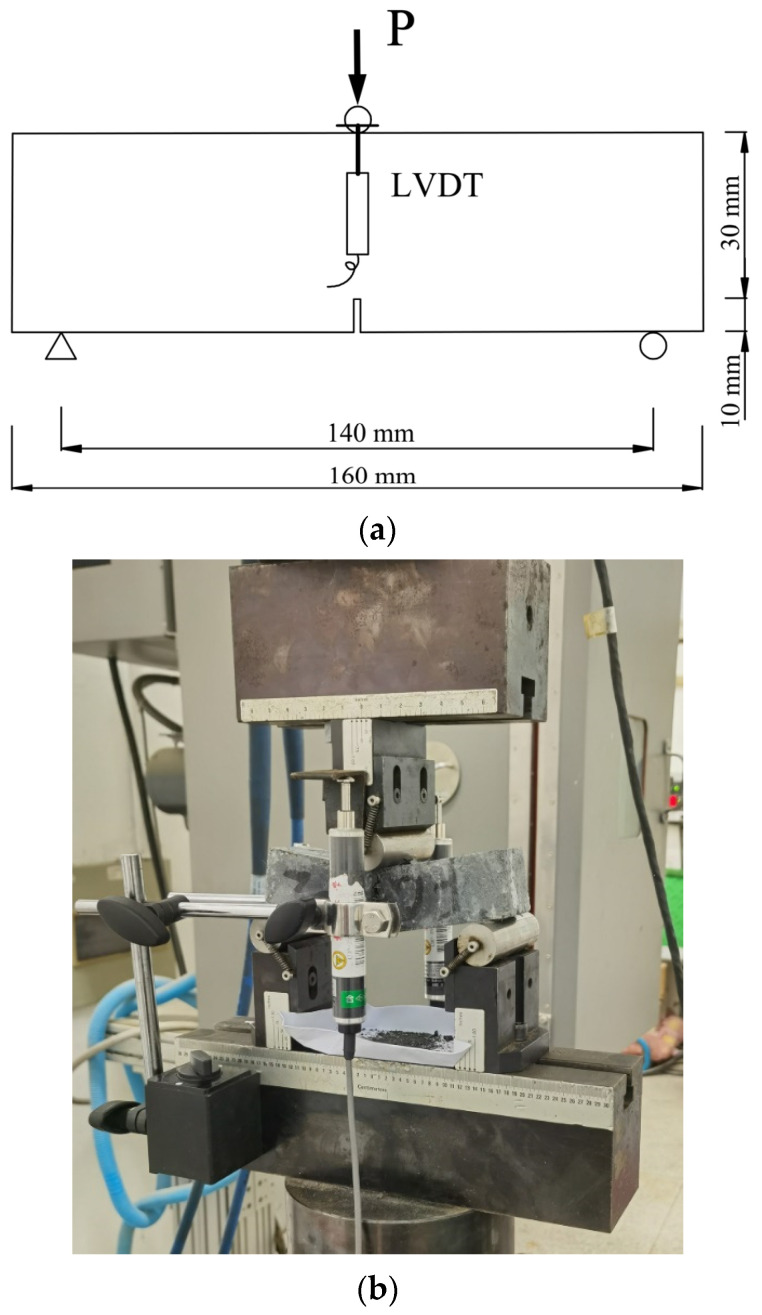
Schematic (**a**) and experimental setup (**b**) of the three-point bending test.

The tested mechanical properties include the compressive and flexural strength at 3 and 28 days and fracture properties at 28 days. The test method for the strength measurements was the same as the method given in standard ISO 679-2009 [21], except that the loading rate of the compressive and flexural strength was 4 kN/s and 100 N/s, respectively. The fracture properties were investigated by conducting a three-point bending test on a notched specimen [9], and three specimens were tested for each group. The notch was prepared at the middle of the specimen using a diamond saw (TDM1260, Bosch, Hangzhou, China) and its depth and width was 10 mm and 3 mm, respectively. The bending test was conducted on an MTS machine (810, MTS, Eden Prairie, MN, USA), and the schematic diagram as well as the experimental setup are given in Figure 2. The span between the two supports was 140 mm, being equal to 3.5 times the depth of the specimen. Two linear variable differential transformers (LVDT) were fixed at the side of the specimen to measure the mid-span deflection, and the measuring capacity of the LVDT was 25 mm. The test was controlled by actuator displacement. The displacement rate was initially 0.5 mm/min until a displacement of 0.5 mm and then was doubled to 1 mm/min for 1 min, followed by 2 mm/min for 1 min, and finally was increased to 5 mm/min for 3 min before the test was ended. Load and deflection data were recorded throughout the loading process and fracture properties, such as peak load, residual load as well as fracture energy, could be analyzed based on the acquired load–deflection (*P–δ*) curves. The fracture energy *G*_F_ was determined according to RILEM TC 50-FMC [22] using the following formula:

(1) $$G_{F}=\left(W_{0}+mg\delta_{0}\right)/A_{\mathrm{lig}}$$

where *W*_0_ is the area enclosed by the *P–δ* curve, *m* is the mass of the specimen between the two roller supports, *δ*_0_ is the deflection at the failure of the specimen, and *A*_lig_ is the area of the initial ligament. In this study, the value of *δ*_0_ is set as 15 mm and the value of *A*_lig_ is 1200 mm^2^.

## 3. Results and Discussion

### 3.1. Flexural Strength

**Figure 3:**
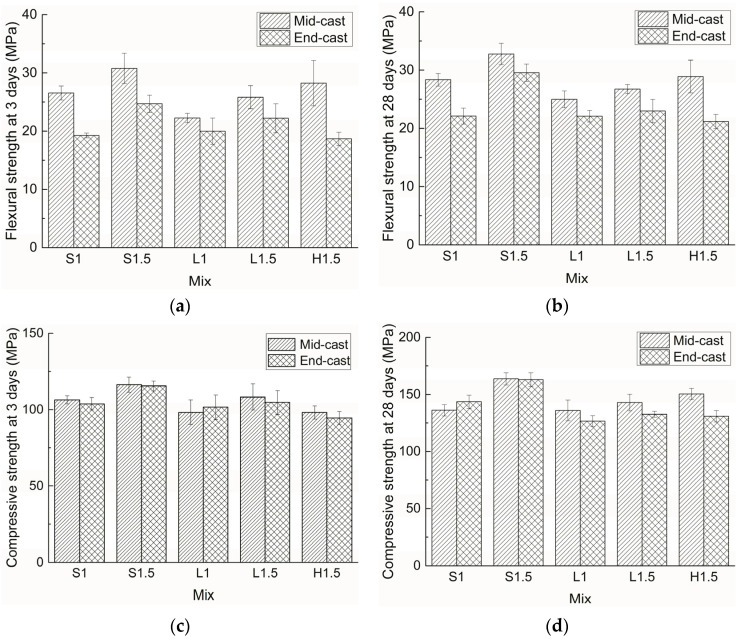
Flexural and compressive strengths of ultra-high performance concrete (UHPC) with two different casting positions: (**a**) flexural strength at 3 days, (**b**) flexural strength at 28 days, (**c**) compressive strength at 3 days, (**d**) compressive strength at 28 days.

The flexural strengths of all the mixes with two different casting positions are given in Figure 3a,b. It can be readily observed that flexural strength increases with fiber content. As the fiber content increases from 1% to 1.5%, the strength gain of the mid-cast specimens is about 15% while that of the end-cast specimens shows great variation, ranging from 4% to 34%. The flexural strength at 3 days is about 90% of that at 28 days, which indicates the formation of a strong fiber–matrix bond at the early age. In addition, specimens with short straight fibers have higher flexural strength than those with long straight fibers or hooked-end fibers.

In Figure 3a,b, it can be seen that the mid-cast specimens show higher flexural strength than their end-cast counterparts. This is mainly because during casting the fibers move more slowly than the mortar and thus, in mid-cast mode, fibers tend to aggregate at the middle of the mold, which leads to an enhanced load-bearing capacity in the flexural test. Compared to specimens with straight fibers, the flexural strength of specimens with hooked-end fibers is more likely to be affected by the casting position. At 28 days, the flexural strengths of S1.5 and L1.5 with end casting are 90% and 86% of their mid-cast counterparts, respectively, while the ratio is merely 73% for H1.5. This could be attributed to the relatively large dimension and irregular shape of the hooked-end fibers, which might encounter more resistance during migration, resulting in a big difference in fiber content at the middle of the specimen using the two casting methods. Moreover, it can be seen that the influence of casting position on flexural strength is affected by fiber content. With the increase of fibers in the material, the flexural strength of specimens with short straight fibers become less affected by the casting position while that of specimens with long straight fibers shows an opposite trend.

### 3.2. Compressive Strength

The compressive strengths of all the mixes with two different casting positions are given in Figure 3c,d. The compressive strength increases with fiber content, and the strength gain is quite different for different samples. For mid-cast specimens, with an increase in fiber content from 1% to 1.5%, the strength gain is about 10% at 3 days and varies from 5% to 20% at 28 days; for end-cast specimens, the strength gain with an increase of fiber varies from 3% to 11% at 3 days, and from 5% to 13% at 28 days. Compared to flexural strength, the strength gain of compressive strength is generally less significant when fiber content increases from 1% to 1.5%. Like the trend observed in flexural strength, the compressive strengths of specimens with short straight fibers are higher than those with the other two fibers. In addition, the compressive strength at 3 days is about 70–80% of that at 28 days, which is lower than the ratio for flexural strength. This is because flexural strength is mainly related to the strength of the fiber and the mechanical bond between the fiber and the matrix, while the compressive strength is mainly governed by the strength of the matrix, which develops with age as the internal pores are progressively filled by hydration products.

Though most mid-cast samples show slightly higher strength than their end-cast counterparts, the influence of casting position on compressive strength is much less significant than that on flexural strength. At 3 days, the strength difference owing to the different casting position is within 5% for all the mixes, and at 28 days the difference is below 8% except for H1.5. Therefore, it can be concluded that casting position has a negligible influence on compressive strength regardless of fiber type and fiber content.

### 3.3. Fracture Properties

**Figure 4:**
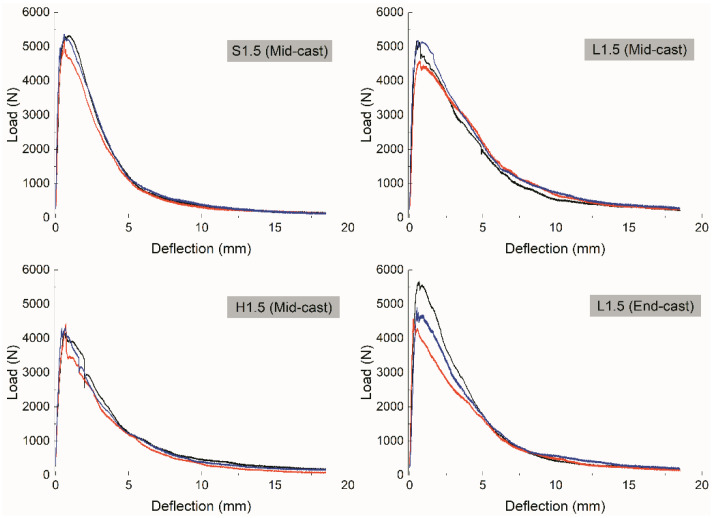
Load–deflection curves of four groups of UHPCs.

The *P–δ* curves of four groups of UHPCs with fiber contents of 1.5% are presented in Figure 4, and all the curves show deflection-hardening behavior. For the first three groups, which are mid-cast samples, the *P–δ* curves were almost superposed on each other, indicating that in the middle cast mode, the fracture property is quite similar among different replicates. The softening curves of UHPCs with straight fibers are relatively smooth, while those with hooked-end fibers show sporadic jags. This could be because, as fibers are pulled out progressively, the bearing capacity of the specimen experiences a sharp drop when the anchoring effects of the bridging hooked-end fibers disappear. Comparing the right two graphs in Figure 4, i.e., the *P–δ* curves of L1.5 with different casting methods, it can be observed that the peak loads of the end-cast samples are scattered to some extent, thus leading to large divergences in the curves. This could be attributed to discrepancies in fiber orientation and distribution at the middle of the specimen among different replicates.

**Figure 5:**
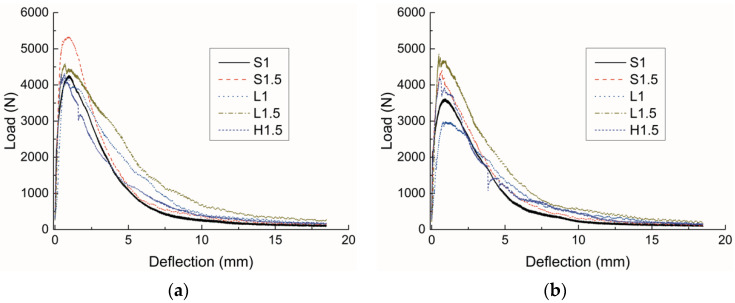
Representative load–deflection curves of all UHPCs with two different casting positions: (**a**) mid-cast, (**b**) end-cast.

**Figure 6:**
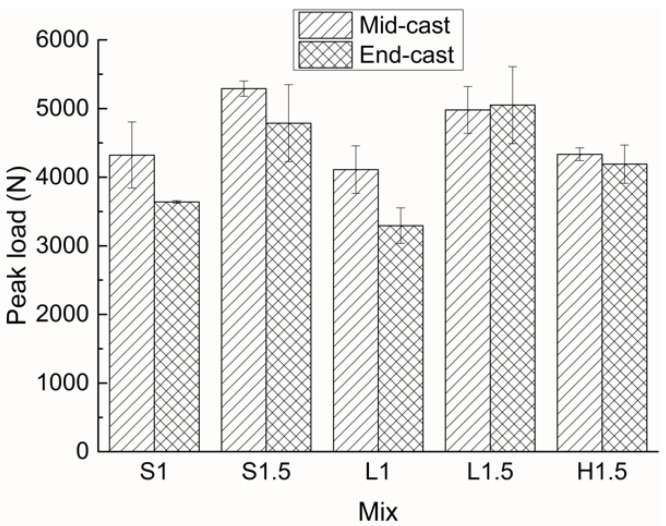
Peak loads of all the UHPCs with two different casting positions.

The representative *P–δ* curves of all the mixes with two different casting positions are given in Figure 5, and the peak loads of all the samples are presented in Figure 6. Similar to the results for flexural strength, specimens with short straight fibers have higher peak loads than those with long straight fibers. However, the latter exhibit higher toughness, which can be characterized by the area under the *P–δ* curve, than the former. This is ‘sbecause the densely distributed short fibers can arrest and delay the formation and coalescence of micro-cracks, which leads to a higher peak load, while the long fibers are more effective in preventing the propagation of macro-cracks and thus contribute to a higher toughness [23]. Compared to specimens with straight fibers, the samples with hooked-end fibers show lower mechanical performance, which is inconsistent with the results in the literature [24,25]. This might indicate that in this work the hooked-end fibers have a poor orientation and distribution at the middle of the specimen, and thus the enhancing effects of the fibers are not well developed.

**Figure 7:**
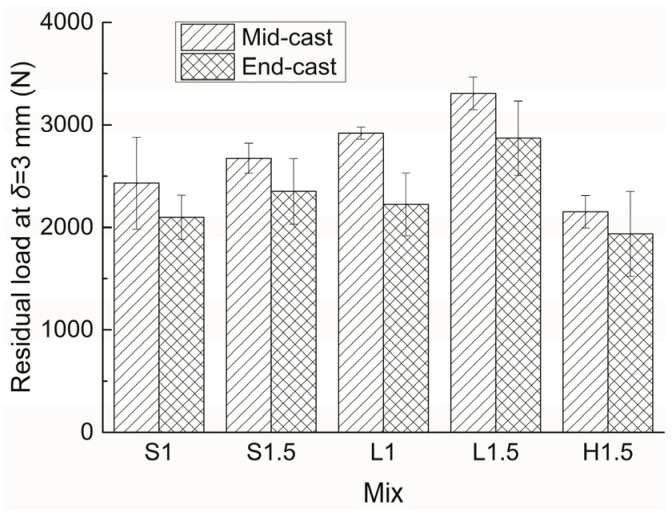
Residual loads of all UHPCs with two different casting positions.

The residual loads at *δ* = 3 mm of all the mixes with two different casting positions are presented in Figure 7. Compared with Figure 3 and Figure 6, it can be seen that the influences of casting position on peak load and residual load are similar to that on flexural strength. Mid-cast specimens generally have higher peak loads and residual loads than their end-cast counterparts. For mix H1.5, the influence of casting position on the two loads is much less significant than that on flexural strength, which again indicates that the distribution of hooked-end fibers varies considerably between different replicates. Moreover, the residual-to-peak load ratio (R/P) can be used to evaluate the post-cracking load-bearing capacity of a structural member. For each mix, the R/P values are close independent of the two casting positions. The R/P value is affected by fiber type, with higher values for long straight fiber reinforced specimens, medium values for short straight fiber reinforced specimens, and lower values for hooked-end fiber reinforced specimens. In addition, the R/P value decreases as fiber content increases. Therefore, among the five mixes, L1 shows the highest R/P value of about 0.7, and H1.5 gives the lowest R/P value of about 0.48.

**Figure 8:**
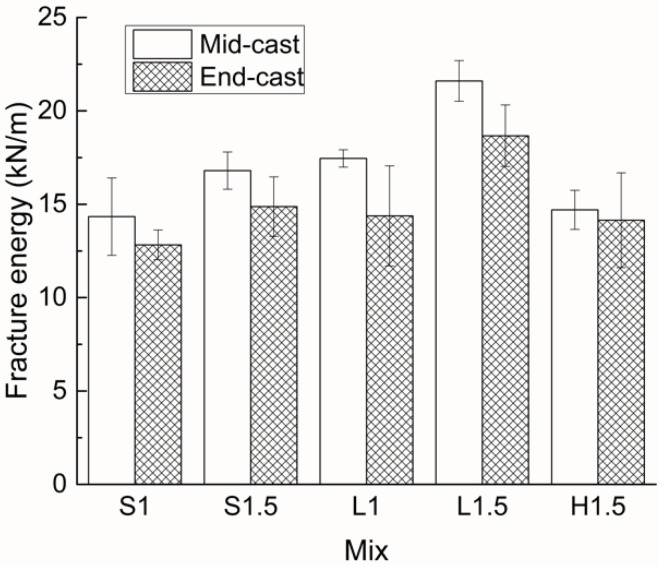
Fracture energies of all UHPCs with two different casting positions.

Figure 8 presents the fracture energies of all the UHPCs with two different casting positions. It can be observed that the basic shape and trend of the bar graph are almost the same as those shown in Figure 7, which implies that the residual load could be used as a simplified toughness index. With the increase of fiber content from 1% to 1.5%, the fracture energy of UHPC is increased by about 20%. The fracture energy of specimens with long straight fibers is higher than that with the other two fibers, and even L1 shows higher fracture energy than S1.5 and H1.5. Compared with end casting, the fracture energy is increased under middle casting. The increase rate is 12% and 20% for short straight and long straight fiber reinforced specimens, respectively, and merely 4% for specimens with hooked-end fibers. In addition, the fracture energy of end-cast samples is generally more scattered than that of mid-cast ones.

## 4. Conclusions

This study investigated the influence of casting position on strength and fracture properties of UHPC with different fiber types and fiber contents. It was found that mid-cast specimens generally show higher flexural strength and fracture properties than end-cast specimens, and that the flexural strength of specimens with hooked-end fibers is more likely to be affected by the casting position. The findings confirm the significance of casting on the mechanical properties of UHPCs, and it is recommended that in order to achieve consistent and reliable experimental results or high-quality industrial products, material manufacturing details, such as casting position, should be seriously taken into account.

## Figures and Tables

**Table 1:** Physical properties and chemical composition of cement and silica fume.

Property	Cement	Silica Fume
Specific surface area, m^2^/kg	362	22,200
Specific gravity	3.1	2.26
SiO_2_, %	21.35	91.2
Al_2_O_3_, %	4.67	2.22
Fe_2_O_3_, %	3.31	0.88
CaO, %	62.6	1.2
MgO, %	3.08	1.25
SO_3_, %	2.25	0.64
Na_2_O, %	0.54	-
K_2_O, %	0.21	-
LOI, %	1.45	2.14

**Table 2:** Dimensions of three types of steel fibers.

Type	Diameter (mm)	Length (mm)	Aspect Ratio
Short straight	0.1	8	80
Long straight	0.2	13	65
Hooked-end	0.34	21	-

**Table 3:** Mix proportion of ultra-high performance concrete (UHPC).

Components of Binder (%)		Aggregate-to-Binder Ratio	w/b	Superplasticizer (% Solid by Weight of Binder)	Fiber (% by Volume)
Cement	Silica Fume				
88	12	1	0.2	0.9	1, 1.5

## Data Availability

The data used to support the findings of this study are available from the corresponding author (Zhao. S) upon request.
